# Overlooked Role of Mesoscale Winds in Powering Ocean Diapycnal Mixing

**DOI:** 10.1038/srep37180

**Published:** 2016-11-16

**Authors:** Zhao Jing, Lixin Wu, Xiaohui Ma, Ping Chang

**Affiliations:** 1 Department of Oceanography, Texas A&M University, College Station, TX77840, USA; 2 Physical Oceanography Laboratory/Qingdao Collaborative Innovation Center of Marine Science and Technology, Ocean University of China, Qingdao, PRC; 3 Texas A&M University, Department of Atmospheric Sciences, College Station, 77843-3146, TX, USA

## Abstract

Diapycnal mixing affects the uptake of heat and carbon by the ocean as well as plays an important role in global ocean circulations and climate. In the thermocline, winds provide an important energy source for furnishing diapycnal mixing primarily through the generation of near-inertial internal waves. However, this contribution is largely missing in the current generation of climate models. In this study, it is found that mesoscale winds at scales of a few hundred kilometers account for more than 65% of near-inertial energy flux into the North Pacific basin and 55% of turbulent kinetic dissipation rate in the thermocline, suggesting their dominance in powering diapycnal mixing in the thermocline. Furthermore, a new parameterization of wind-driven diapycnal mixing in the ocean interior for climate models is proposed, which, for the first time, successfully captures both temporal and spatial variations of wind-driven diapycnal mixing in the thermocline. It is suggested that as mesoscale winds are not resolved by the climate models participated in the Coupled Model Intercomparison Project Phase 5 (CMIP5) due to insufficient resolutions, the diapycnal mixing is likely poorly represented, raising concerns about the accuracy and robustness of climate change simulations and projections.

Diapycnal mixing in the ocean influences vertical transport of heat, dissolved gases, nutrients, and pollutants. Understanding its spatial and temporal variation is a key to improving model representation and prediction of large-scale ocean circulation and climate^1^,^2^,^3^,^4^,^5^,^6^,^7^,^8^. Much of the diapycnal mixing in ocean interior occurs through internal wave breaking^1^. Energy is input into the internal wave field primarily through surface wind forcing and interactions of ocean bottom flow with topography^1^. As a natural resonant frequency of flows on a rotating planet, near-inertial internal waves (NIWs) are efficiently forced by fluctuating wind stresses^9^,^10^. They form a pronounced peak in energy spectrum of ocean currents, containing half of the kinetic energy in the internal wave field and a substantial portion of the shear variance^10^. Extensive observations suggest that wind-generated NIWs play a key role in furnishing the diapycnal mixing in the upper ocean, dictating its spatial and temporal variability^11^,^12^,^13^,^14^. In particular, the diapycnal mixing in the upper 1000 m is significantly enhanced in the Kuroshio extension region due to the strong NIWs there^11^,^13^.

However, important influences of wind-generated NIWs on diapycnal mixing in the thermocline are not properly accounted for in the current generation of climate models. For instance, to the best of our knowledge, the elevated diapycnal mixing observed in the Kuroshio extension region has not been replicated by any state-of-the-art ocean or climate models even though various parameterization schemes have been applied^15^,^16^,^17^,^18^,^19^,^20^,^21^,^22^,^23^. This might be partly due to inadequacies in the parameterization schemes. In addition, it raises a question whether the contribution of near-inertial energy to diapycnal mixing is severely underestimated by present climate models. In fact, previous studies suggest that model simulated near-inertial kinetic energy is significantly weaker than the observed estimate derived from surface drifters^21^,^24^,^25^,^26^,^27^,^28^. One possible cause is the lack of contribution from mesoscale winds to NIW generation due to the coarse (>1°) horizontal resolution of surface wind forcing in climate models. Here mesoscale is defined as scales of a few hundred kilometers. The mesoscale winds are mainly associated with midlatitude atmospheric fronts and tropical cyclones. Both are efficient generators of NIWs^29^. In this study, we investigate the role of mesoscale winds in powering diapycnal mixing in the thermocline.

## Results

To understand influences of mesoscale winds on NIWs and diapycnal mixing, we analyze simulation results derived from a high-resolution coupled regional climate model (CRCM)^30^. The CRCM is configured over the North Pacific, where extensive midlatitude winter storms and tropical cyclones prevail in winter and summer, respectively. Both the oceanic and atmospheric model components have a horizontal resolution of ~0.1°, so that mesoscale oceanic eddies and winds are well resolved. The simulation analyzed was initialized on October 1, 2002 and integrated for one year. The period 2002–2003 is chosen as it is a relatively neutral year for the Pacific Decadal Oscillation (PDO) that modulates midlatitude storms activities^31^. This facilities comparisons of the simulated NIW strength to the observed climatological mean derived from surface drifters^27^,^28^. To isolate the contribution of mesoscale winds to near-inertial energy flux, we further perform an uncoupled ocean simulation using the same ocean model, and the simulated winds from the CRCM but spatially smoothed and then subsampled onto a 2.5° × 2.5° grid. This simulation is referred to as the Ocean_2.5 in which mesoscale winds are filtered out. Readers are referred to *Ma et al.*^30^ and the method section for detailed description of CRCM and Ocean_2.5 simulations.

**Figure 1:**
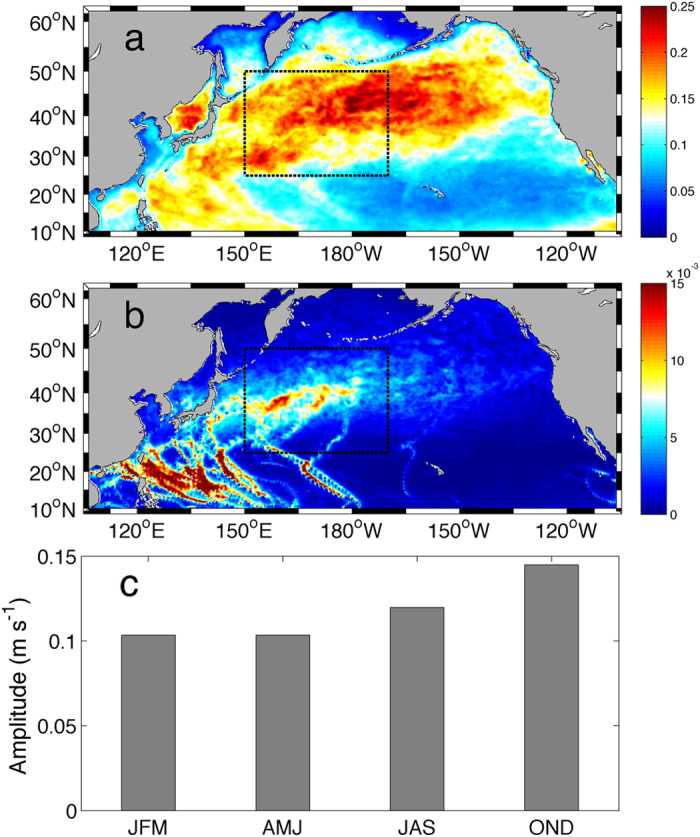
Simulated near-inertial motions in CRCM. The annual spatial distribution of (**a**) surface near-inertial current amplitude (m s^−1^) and (**b**) near-inertial energy flux input by winds (W m^−2^) simulated in CRCM. Note that the colorbar of (b) is saturated. (**c**) The seasonal cycle of surface near-inertial current amplitude over the North Pacific simulated in CRCM. The maps were generated using M_Map V1.4 package for Matlab (http://www.eos.ubc.ca/~rich/map.html).

**Figure 2:**
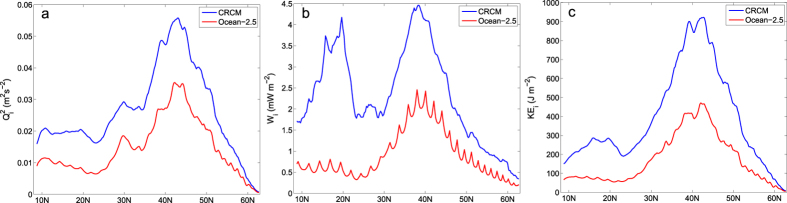
The latitudal dependence of annual mean (**a**) surface near-inertial current variance 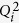, (**b**) near-inertial energy flux *W*_*i*_, and (**c**) near-inertial kinetic energy density in the surface boundary layer *KE*_*i*_. The blue and red lines correspond to the values in CRCM and Ocean_2.5, respectively.

The surface near-inertial currents simulated by CRCM show good agreements with the observations^27^,^28^. The mean near-inertial current amplitude over the North Pacific is about 12.3 cm s^−1^ (Fig. 1a), comparable to 11.5 cm s^−1^ obtained from surface drifters^27^. The most energetic near-inertial currents are found around 43°N collocated with the North Pacific storm track (Fig. 2a). The zonal-mean near-inertial current variance there reaches up to 55 cm^2^ s^−2^ and is close to the observed value (~50 cm^2^ s^−2^)^28^. The seasonal cycle of the simulated near-inertial motions (Fig. 1c) is also consistent with the observed estimate^27^. Both exhibit the strongest near-inertial current during October-December and a second maximum during July-September. These agreements provide credibility of the simulated near-inertial energy flux from the atmosphere into the ocean in CRCM.

In CRCM, the near-inertial energy flux input by winds exhibits pronounced peaks in the latitudal bands of 30°N–50°N and 10°N–20°N (Fig. 2b), respectively. The peak within 30°N–50°N is mainly due to the strong near-inertial energy flux input by atmospheric fronts and synoptic storms over the Kuroshio extension region (Fig. 1b). The sharp sea surface temperature front there plays an important role in the maintenance of near-surface baroclinicity of atmosphere^32^,^33^, which is a key to the development of atmospheric fronts and synoptic storms during boreal winter. The peak within 10°N–20°N, on the other hand, is related to the energetic tropical cyclones, as evidenced by the pronounced near-inertial energy flux injected along the paths of individual tropical cyclones (Fig. 1b).

When mesoscale winds are removed in Ocean_2.5, both these peaks are significantly reduced (Fig. 2b). In particular, the near-inertial energy flux peak within 10°N–20°N almost disappears because tropical cyclones are filtered out in the Ocean_2.5. The total near-inertial energy flux integrated over the North Pacific is around 0.16 TW in CRCM, but decreases to about 0.05 TW in Ocean_2.5. Due to the reduced near-inertial energy flux into the ocean, the NIWs in Ocean_2.5 become much weaker than those in CRCM (Fig. 2c). The mean near-inertial kinetic energy density in the surface boundary layer is 400 J m^−2^ in CRCM, more than twice the value of 175 J m^−2^ in Ocean_2.5.

The above analyses suggest that the mesoscale winds account for the bulk of near-inertial energy flux into the ocean and may thus play an important role in powering diapycnal mixing in the thermocline. To quantify the contribution of mesoscale winds to diapycnal mixing in the thermocline, a modified version of the finescale parameterization^34^ (MFP) is developed in this study to compute the diapycnal mixing offline using the data from CRCM and Ocean_2.5 simulations (See the method section for details). The MFP relates turbulent kinetic dissipation rate to downscale energy cascade of internal waves in vertical wavenumber space that depends on latitude, background stratification, near-inertial shear variance and vertical grid size in models. Its validity is justified by its successful application to the Moored Profiler data in the Kuroshio extension region collected during the Kuroshio Extension System Study (KESS) program (See Supplementary Figure S1).

**Figure 3:**
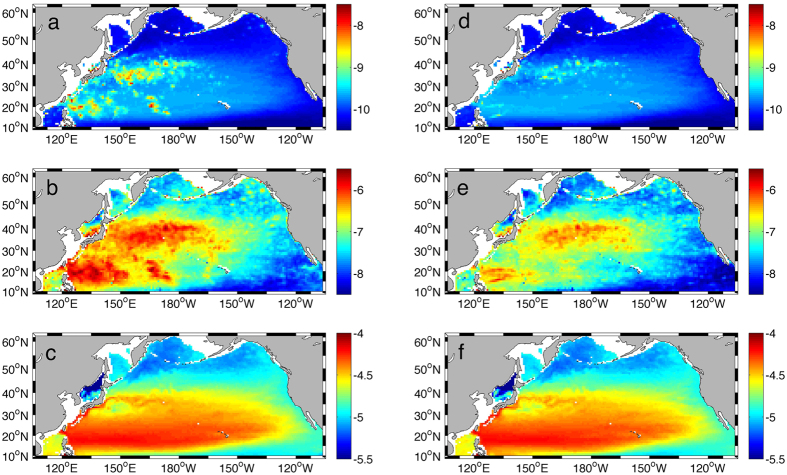
The annual spatial distribution of diapycnal mixing parameterized by the MFP in CRCM and Ocean_2.5. (**a**–**c**) show the parameterized dissipation rate log_10_ *ε* in m^2^ s^−3^, near-inertial shear variance log_10_ 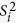 in s^−2^, and background stratification log_10_ *N*^2^ in s^−2^ within 250–500 m in CRCM, respectively. (**d**–**f**) show the values in Ocean_2.5. The maps were generated using M_Map V1.4 package for Matlab (http://www.eos.ubc.ca/~rich/map.html).

In CRCM, the parameterized turbulent kinetic dissipation rate *ε*_*C*_ within the thermocline (250–500 m) shows pronounced spatial variability (Fig. 3a) and agrees reasonably well with the observations^11^,^12^. The Northeast Pacific and subpolar North Pacific are associated with weak diapycnal mixing with *ε*_*C*_ less than 1 × 10^−10^ m^2^ s^−3^. Enhanced *ε*_*C*_ is localized in the Kuroshio extension region and subtropical Northwest Pacific where its value varies from 1 × 10^−9^ to 1 × 10^−8^ m^2^ s^−3^. Significant discrepancy between simulated and observed dissipation values is confined mainly to the regions with rough topography (e.g., the Hawaiian Ridge and Izu-Bonin-Mariana Arc). In these regions, the diapycnal mixing is not wind-driven but likely furnished by energetic internal tides generated through interactions of barotropic tides with bottom topography^1^, which is not included in the CRCM simulation.

**Figure 4:**
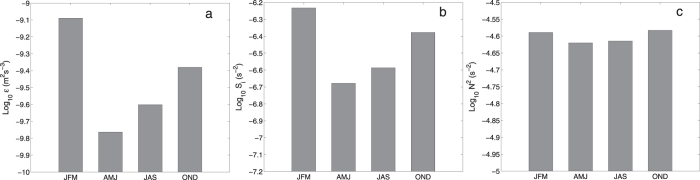
The seasonal cycle of diapycnal mixing parameterized by the MFP in the CRCM. (**a**–**c**) show the parameterized dissipation rate log_10_ *ε* in m^2^ s^−3^, near-inertial shear variance log_10_ 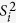 in s^−2^, and background stratification log_10_ *N*^2^ in s^−2^ within 250–500 m in the region 25°N–50°N, 150°E-170°W (black box in Fig. 1a and b) in CRCM, respectively.

In addition to the pronounced spatial variability, *ε*_*C*_ exhibits a clear seasonal cycle. Figure 4a displays the mean *ε*_*C*_ between 150°E-170°W, 25°–50°N in different seasons. It is significantly enhanced in winter with values during October-March about three times of those during April-September. This seasonal variation is close to the observed estimate^11^,^13^ in the same region, giving further support that *ε*_*C*_ is a good parameterization of wind-driven diapycnal mixing in the thermocline.

The spatial distributions of *ε*_*C*_ and wind-input near-inertial energy flux in CRCM agree well with each other (Figs 1b and 3a), suggesting that the wind-generated NIWs may play a dominant role in controlling the variability of diapycnal mixing in the thermocline. To further demonstrate this point, we examine the relation of *ε*_*C*_ to the near-inertial shear variance 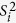 and background stratification *N*^2^, both of which have a direct influence on the value of *ε*_*C*_. In CRCM, the spatial correlation between *ε*_*C*_ and 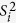 is about 0.83 (*p* value <0.01 based on a t-test) while it decreases to 0.68 between *ε*_*C*_ and *N*^2^ (*p* value <0.01). In particular, although the strong stratification extends to the whole subtropical gyre, the enhanced *ε*_*C*_ is well confined to the Kuroshio extension region and subtropical Northwest Pacific associated with high 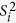 (Fig. 3a–c). The NIW activity not only dominates the spatial variability of *ε*_*C*_ but also its seasonal cycle. In the region between 150°E-170°W and 25°–50°N, both 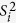 and *ε*_*C*_ exhibit significant enhancement in winter (Fig. 4a,b). In contrast, *N*^2^ varies by less than 10% seasonally (Fig. 4c). In other words, wind-generated NIWs play a key role in modulating the spatial and temporal variations of diapycnal mixing in the thermocline in the North Pacific.

In Ocean_2.5, 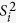 is significantly reduced due to the decreased near-inertial energy flux into the ocean, which significantly weakens the diapycnal mixing in the thermocline (Fig. 3d,e). The mean dissipation rate within 250–500 m in the North Pacific is 3.1 × 10^−10^ m^2^ s^−3^ in CRCM, more than twice the value of 1.4 × 10^−10^ m^2^ s^−3^ in Ocean_2.5. In particular, the dissipation rate in Ocean_2.5 does not exhibit enhancement in the subtropical Northwest Pacific because most of the near-inertial energy flux there is injected by tropical cyclones which are filtered out in Ocean_2.5. Furthermore, although the dissipation rate in Ocean_2.5 is slightly enhanced in the Kuroshio extension region due to the strong near-inertial energy flux input by synoptic winter storms, the value there is generally lower than 5.0 × 10^−10^ m^2^ s^−3^. Such an enhancement is significantly weaker compared to that in CRCM (Fig. 3a,d) and observations^11^,^12^, suggesting the important role of mesoscale winds in powering the diapycnal mixing in the Kuroshio extension region.

## Discussion

While tidal mixing has been relatively well represented in present climate models^16^,^17^,^18^,^19^,^20^, parameterization of wind-driven mixing remains to be a great challenge. In this study, we developed and tested a new parameterization (MFP) of wind-driven diapycnal mixing in ocean interior for climate models. Both the temporal and spatial variations of wind-driven diapycnal mixing parameterized by the MFP show good agreement with those derived from observations in the thermocline of North Pacific. In addition to the improvement in the MFP compared to the previous parameterization schemes, the results reveal a key role of mesoscale winds in powering diapycnal mixing through generation of energetic NIWs. Without the contribution of mesoscale winds, the diapycnal mixing parameterized by the MFP differs significantly from the observations.

Currently, most of the climate models in the Coupled Model Intercomparison Project Phase 5 (CMIP5)^35^ are not able to resolve mesoscale winds due to their coarse atmospheric resolution (>1°), causing great uncertainties in the model parameterized diapycnal mixing in the thermocline. It remains unclear how these uncertainties may affect the ocean circulation and stratification. Several sensitivity tests have been done in previous studies^6^,^7^,^8^, suggesting that the geography of diapycnal mixing in the thermocline has a significant impact on ocean circulations, water properties, and fluxes of heat. However, it should be noted that diapycnal diffusivity values in these sensitivity studies are prescribed in a highly crude way so that the effect of wind-driven diapycnal mixing might not be properly represented. To better evaluate the influence of wind-driven diapycnal mixing on ocean circulations and climate, it may be highly desirable to implement the MFP to climate models, which will be our next step in future studies.

Future studies also need to extend the study domain from current North Pacific region to other ocean basins. Given the similar atmospheric environment to that of North Pacific, it is likely that the major conclusions of this study could extend to the North Atlantic. However, whether the major conclusions in this study also hold in the Southern Ocean – another potentially important region for wind-driven diapycnal mixing – remains undetermined and further tests are needed.

## Methods

### The high-resolution coupled regional climate model (CRCM)

A coupled regional climate model (CRCM) developed at Texas A&M University is used to simulate NIWs in the North Pacific^30^. It includes Weather Research and Forecasting (WRF) Model as the atmospheric component and Regional Ocean Modeling System (ROMS) as the oceanic component. CRCM is configured over the entire North Pacific (See Supplementary Figure S2) with a horizontal resolution of ~0.1° for both WRF and ROMS. There are 30 and 50 vertical levels for WRF and ROMS, respectively. As ROMS is a terrain-following model, its vertical grid size depends on the bathymetry. Figure S3 displays the distribution of vertical grids with a bottom depth of 4500 m (the mean bottom depth of North Pacific basin). There are 19 vertical levels in the upper 200 m. In the thermocline (250–500 m), the vertical grid size ranges from ~15 m to ~45 m.

The CRCM simulation was initialized on October 1, 2002 and integrated for one year. The open boundaries of the ocean are forced by 5-day average Simple Ocean Data Assimilation (SODA) dataset. The initial condition of the ocean is derived from a 6-year spin-up run forced by observationally based version 2 forcing for Coordinated Ocean-ice Reference Experiments (CORE-II). The 6-hourly National Centers for Environmental Prediction (NCEP) reanalysis data are used as the initial and boundary conditions for the atmosphere. In the simulation, WRF and ROMS are coupled hourly, which is sufficiently fine to resolve wind stress variance in the near-inertial band.

The thermocline stratification in the CRCM is comparable to that derived from World Ocean Atlas 2009 (See Supplementary Figure S4). In addition, the simulated mixed layer depth *MLD*_*CRCM*_ is in broad agreement with IFREMER/LOS Mixed Layer Depth Climatology *MLD*_*obs*_^36^ (See Supplementary Figure S5). Their difference, defined as 2(*MLD*_*CRCM*_ − *MLD*_*obs*_)/(*MLD*_*CRCM*_ + *MLD*_*obs*_), is in general within 60% in the North Pacific basin. In particular, in our interested region with strong diapycnal mixing induced by NIWs (i.e., 30°N–45°N, 150°E–180°E and 10°N–30°N, 120°E–150°E), the difference between area-averaged *MLD*_*CRCM*_ and *MLD*_*obs*_ is less than 20%. Finally, the simulated 850-hpa and sea surface storm tracks also agree reasonably well with those derived from the ERA-Interim reanalysis and are close to their climatological mean values (See Supplementary Figure S6). These agreements provide support for the reliability of the CRCM simulation.

An uncoupled ocean simulation using ROMS, forced by the atmospheric surface variables derived from the CRCM simulation, is conducted in the same region within the same period. The surface heat flux and fresh water flux remain unchanged. But the winds are smoothed using a ~2.5° × 2.5° moving average and then subsampled onto ~2.5° horizontal grids. This experiment is referred to as Ocean_2.5. As the only difference between the CRCM and Ocean_2.5 simulations is the lack of mesoscale winds in the Ocean_2.5 simulation, comparisons between the CRCM and Ocean_2.5 simulations provide a rational way to evaluate the role of mesoscale winds in powering oceanic NIWs and diapycnal mixing.

### Computation of near-inertial energy flux, current variance and shear variance

In this study, the near-inertial current (*u*_*i*_, *v*_*i*_) is retained by high-pass filtering the horizontal velocity with a cutoff frequency of 0.8 *f* where *f* is the local Coriolis frequency. Here a 20th-order finite-impulse-response filter based on the Hamming window is used. Further increasing the order of the filter does not make any substantial impact on *u*_*i*_ and *v*_*i*_. The near-inertial current variance and shear variance are defined as 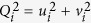 and 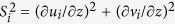, respectively. The near-inertial energy flux is defined as 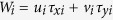 where *τ*_*xi*_ and *τ*_*yi*_ are the high-pass filtered zonal and meridional wind stress components.

### A modified version of finescale parameterization (MFP)

In the finescale parameterization^34^, the turbulent kinetic dissipation rate is parameterized in terms of internal wave shear as:


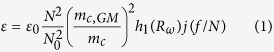


where *m* is the vertical wavenumber, *N* the background buoyancy frequency, *f* the Coriolis frequency, *ε*_0_ = 6.7 × 10^−10^ m^2^ s^−3^, *N*_0_ = 5.2 × 10^−3^ rad s^−1^, and *R*_*ω*_ the shear/strain variance ratio fixed at 3 based on the observations^12^. *h*_1_(*R*_*ω*_) and *j(f*/*N*) are corrections for the frequency content of internal wave field^34^. *m*_*c*_ is a high wavenumber limit representing a transition to the wave-breaking regime and is computed as^34^:


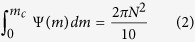


where Ψ(*m*) is the shear spectrum of internal waves. For the Garret-Munk (GM) spectrum^34^, *m*_*c*, *GM*_ is equal to 2*π*/10 rad m^−1^.

The validity of Eqs 1 and 2 has been tested in the open ocean including the Kuroshio extension region^12^,^37^. The values computed from Eqs 1 and 2 agree with those derived from the microstructure measurements within a factor of 2–3. However, Eq. 2 is not directly applicable to climate models because the insufficient vertical resolution of models may significantly underestimate Ψ(*m*). The underestimation results from two aspects. First, most models (e.g., ROMS) solve fluid motions using a finite difference method which assumes a piecewise linear functional form for each physical quantity such as *u* and *v*. This underestimates Ψ(*m*) by a factor of 
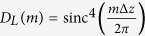
 where 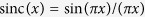 and Δ*z* is the grid size. Second, computation of vertical shear using a first-order difference leads to a further underestimation of Ψ(*m*) by a factor of 
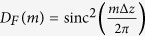
. Therefore, the internal wave shear variance computed from numerical models is equal to:


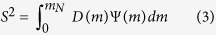


where 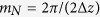 is the Nyquist wavenumber and 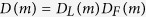.

Previous studies^13^,^38^ suggest that the internal wave shear spectrum is almost flat within m_*_ − *m*_*c*_ and then rolls off roughly as *m*^−1^. Here m_*_ is the spectral bandwidth and may vary from one region to another. Without reliable estimates for m_*_, we approximate m_*_ as zero because m_*_ is typically much smaller than *m*_*c*_. This suggests that Ψ(*m*) can be approximated as:


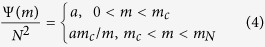


where *a* is a constant. As this approximate form of Ψ(*m*) should also satisfy Eq. (2) for consistency, *a* is equal to 2*π*/(10*m*_*c*_).

By substituting Eq. (4) into Eq. (3), it is evident that the right-hand side of Eq. (3) is a function of *N*^2^ and *m*_*c*_. Given *N*^2^ and *S*^2^, *m*_*c*_ can thus be uniquely determined from Eq. (3).

It should be noted that using m_*_ = 0 tends to overestimate *m*_*c*_. In the depth range 250–500 m where the mean Δ*z* in the CRCM is around 30 m, sensitivity test suggests that *m*_*c*_ varies only within a factor of 1.6 for m_*_ ranging from 0 to 0.02 rad m^−1^ (See Supplementary Figure S7) (The canonical GM spectrum assumes a value of ~0.01 rad m^−1^ for m_*_ in the thermocline ref. 34). Therefore, the uncertainties in dissipation rates from using m_*_ = 0 are probably within a factor of 2.5 and are thus acceptable.

In this study, we use Eqs 1 and 3 to estimate the diapycnal mixing. As we focus on the contribution of wind-generated NIWs to diapycnal mixing, *S*^2^ is replaced by 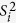. The validity of Eqs 1 and 3 with Δ*z* = 30 m and 50 m is tested using the mooring data collected during the Kuroshio Extension System Study (KESS). Δ*z* = 30 m is comparable to the mean vertical grid size in the thermocline (250–500 m) while Δ*z* = 50 m is close to the coarsest grid size in the thermocline. As shown in Supplementary Figure S1, the dissipation rates estimated from Eqs 1 and 3 agree well with those derived from Eqs 1 and 2, justifying the validity of MFP. Finally, it should be noted that the skill of MFP tends to degrade with the increasing vertical grid size (Supplementary Figure S1). Application of MFP to numerical models with a vertical resolution coarser than that of CRCM should be taken with caution.

## Additional Information

**How to cite this article**: Jing, Z. *et al.* Overlooked Role of Mesoscale Winds in Powering Ocean Diapycnal Mixing. *Sci. Rep.* **6**, 37180; doi: 10.1038/srep37180 (2016).

**Publisher’s note**: Springer Nature remains neutral with regard to jurisdictional claims in published maps and institutional affiliations.

## Supplementary Material

Supplementary Information
