# Supplementary material for: Overlooked Role of Mesoscale Winds in Powering Ocean Diapycnal Mixing
Source: Sci Rep. 2016 Nov 16;6:37180. doi: 10.1038/srep37180 (PMC5111103; doi:10.1038/srep37180)
Supplement: Supplementary Information [file srep37180-s1.doc]

Overlooked Role of Mesoscale Winds in Powering

Ocean Diapycnal Mixing

Zhao Jing^1,2,*^, Lixin Wu^2^, Xiaohui Ma^1,2^, and Ping Chang^1,2,3^

^1^Department of Oceanography, Texas A&M University, College Station, TX77840, USA

^2^Physical Oceanography Laboratory/Qingdao Collaborative Innovation Center of Marine Science and Technology, Ocean University of China, Qingdao, PRC

3. Texas A&M University, Department of Atmospheric Sciences, College Station, TX, US, 77843-3146

*Corresponding author E-mail: jingzhao198763@tamu.edu


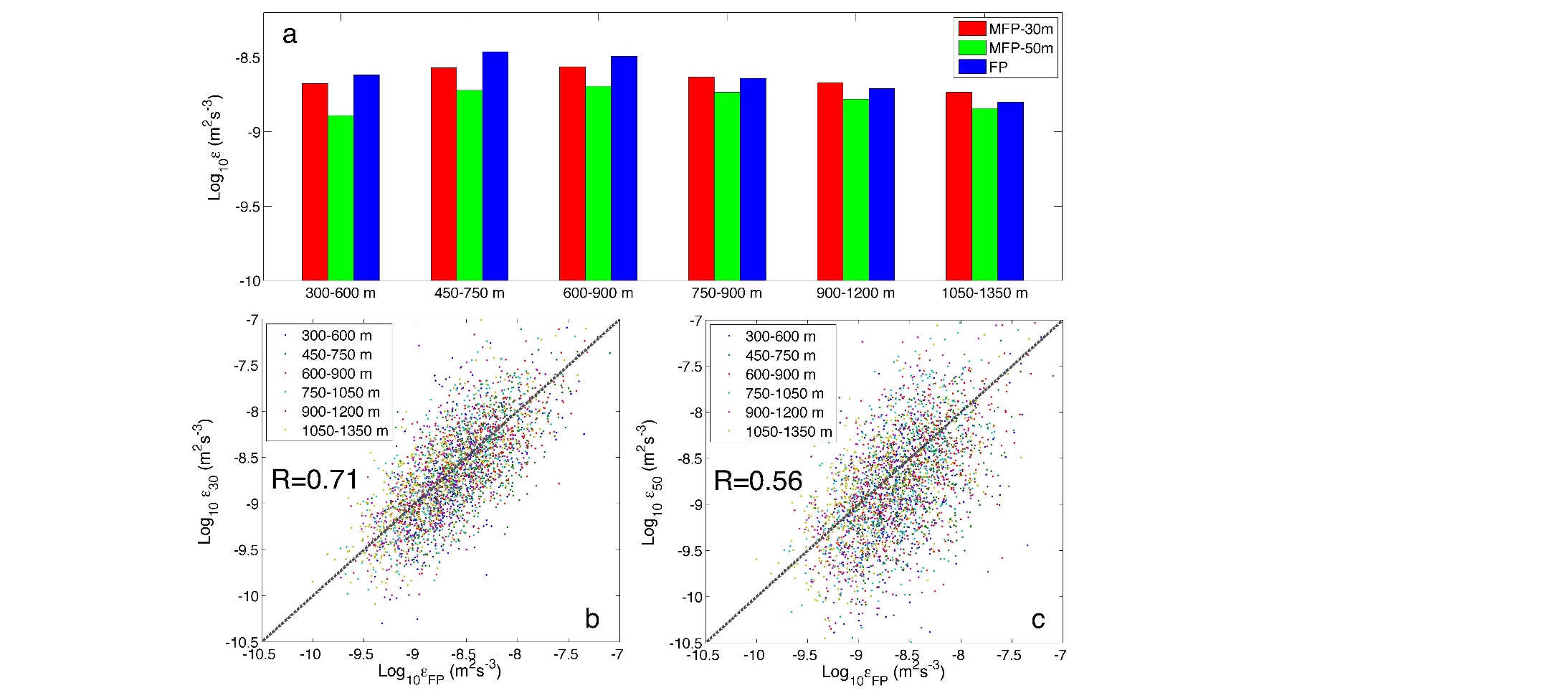


Figure S1 The comparison between dissipation rates computed from the finescale parameterization (FP) and modified finescale parameterization (MFP) based on the McLane Moored Profiler (MMP) data collected during Kuroshio Extension System Study (KESS). The vertical resolution of horizontal velocity measured by MMP is 2 m. For the MFP method, the velocity data are first convolved with a $2\Delta z$-wide triangular function of which the spectral transfer function in the vertical wavenumber domain is $D_{L}(m)$. Then we subsample the data on coarser grids with an interval of $\Delta z$ and compute the vertical shear using the first-order difference. In this case,$D(m)=D_{F}(m)D_{L}(m)$. (a) The time-mean dissipation rate at different depth intervals derived from the FP (blue) and MFP with $\Delta z$=30 m (red) and $\Delta z$=50 m (green). (b) The scatterplot of instantaneous dissipation rates (colored dots) derived from the FP *vs.* MFP with $\Delta z$=30 m. *R* denotes the correlation coefficient. (c) Similar to (b) but for the FP *vs.* MFP with $\Delta z$=50 m.


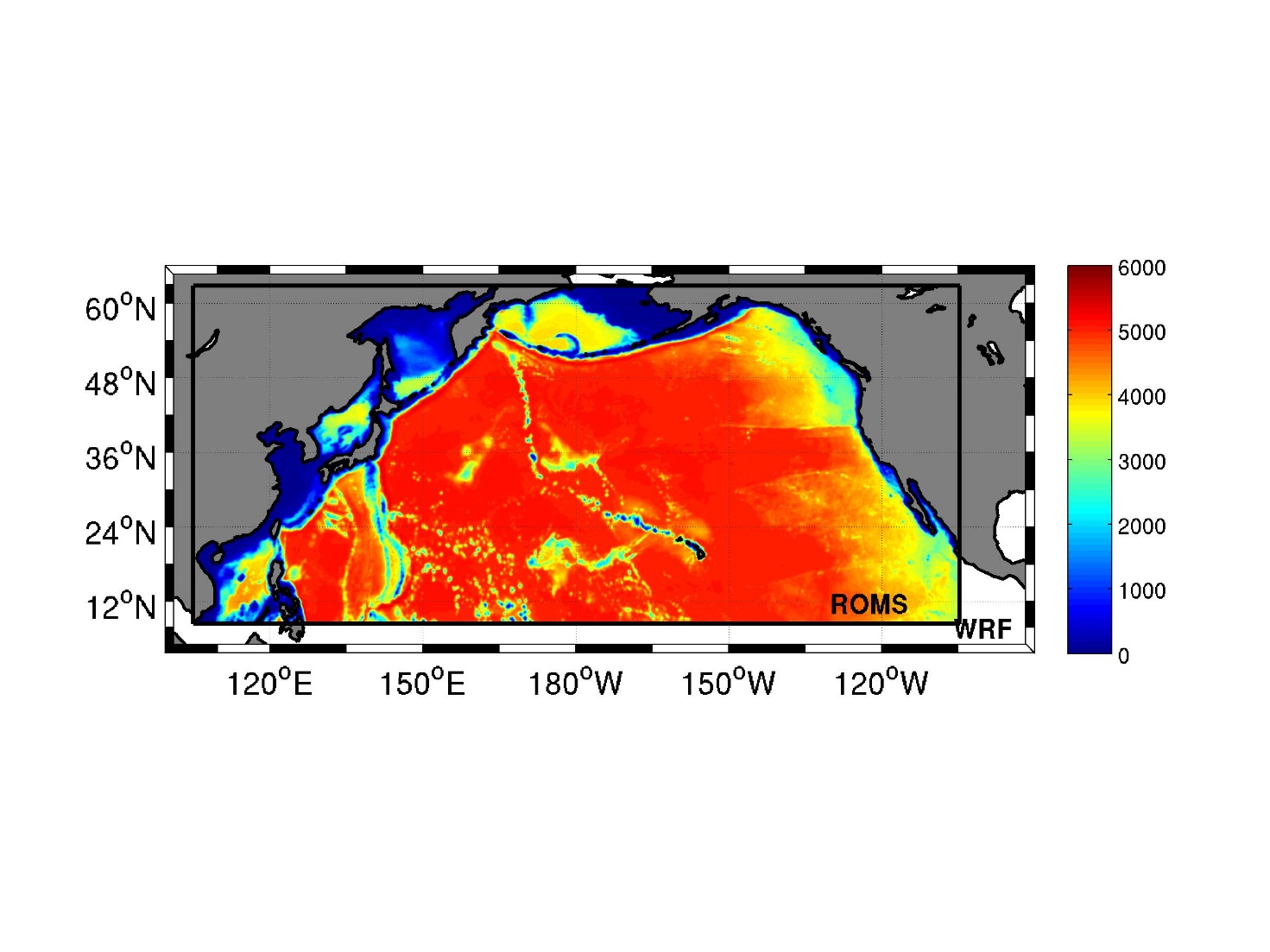


Figure S2 CRCM computation domain in the North Pacific: the outer frame outlines the WRF computational region and the inner frame outlines the ROMS computational region. The colorbar represents the topography. The maps were generated using M_Map V1.4 package for Matlab (http://www.eos.ubc.ca/~rich/map.html).


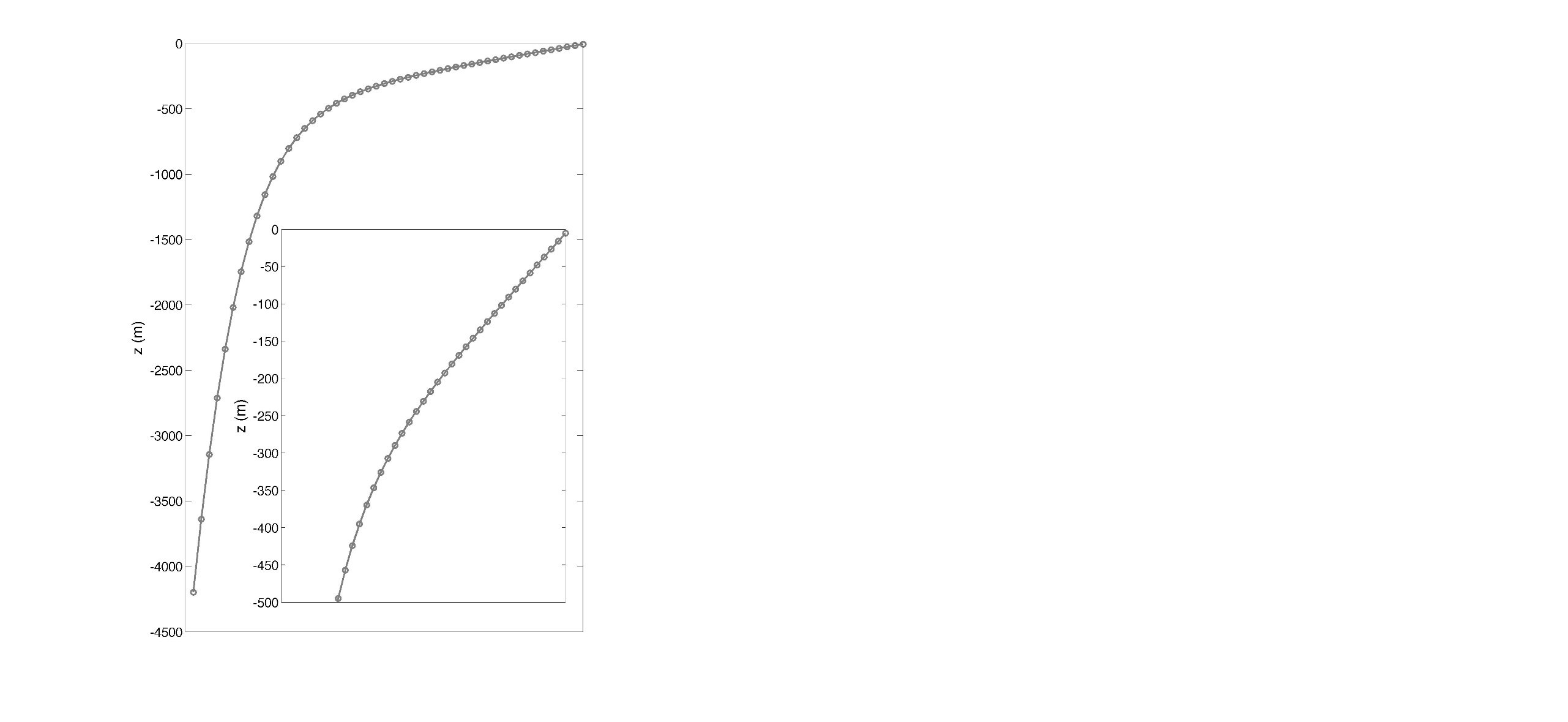


Figure S3 Distribution of vertical grids in ROMS with a bottom depth of 4500 m


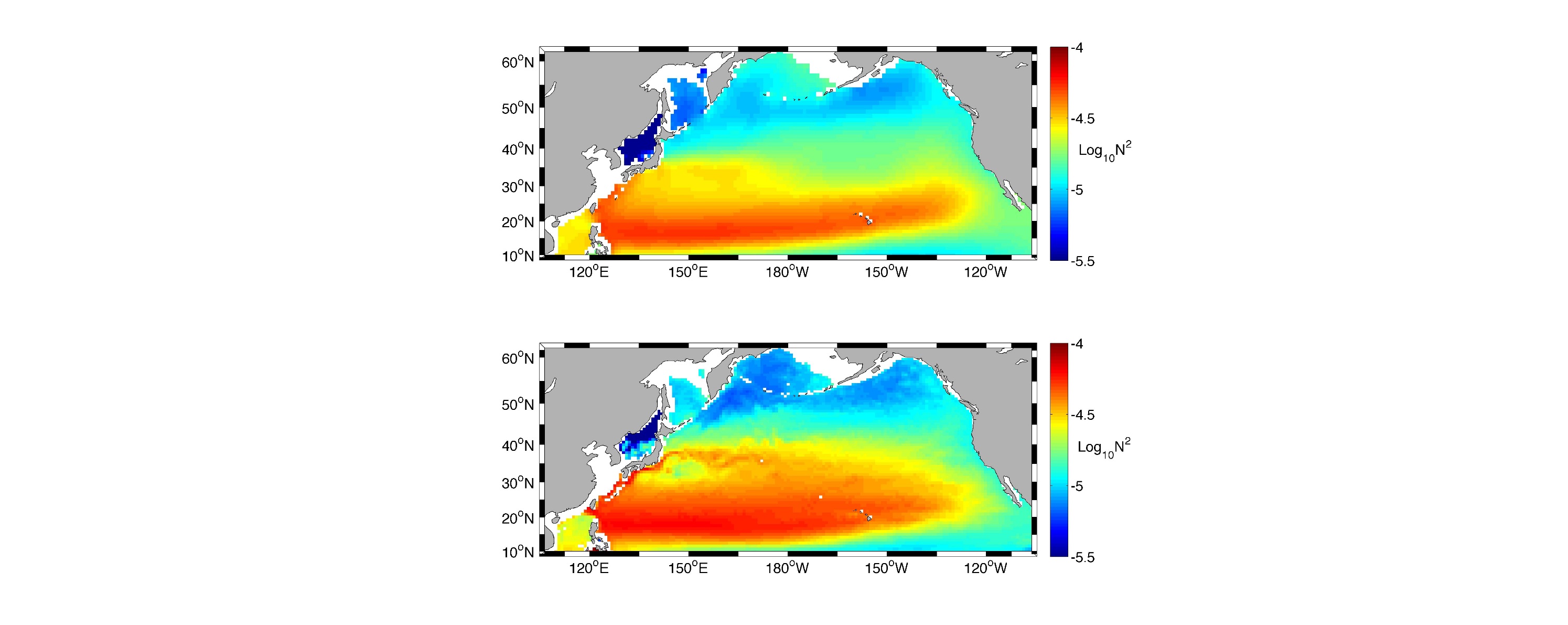


Figure S4 The mean stratification $N^{2}$within 250-500 m computed from the WOA2009 (upper panel) and CRCM (lower panel). The unit here is s^-2^. The maps were generated using M_Map V1.4 package for Matlab (http://www.eos.ubc.ca/~rich/map.html).


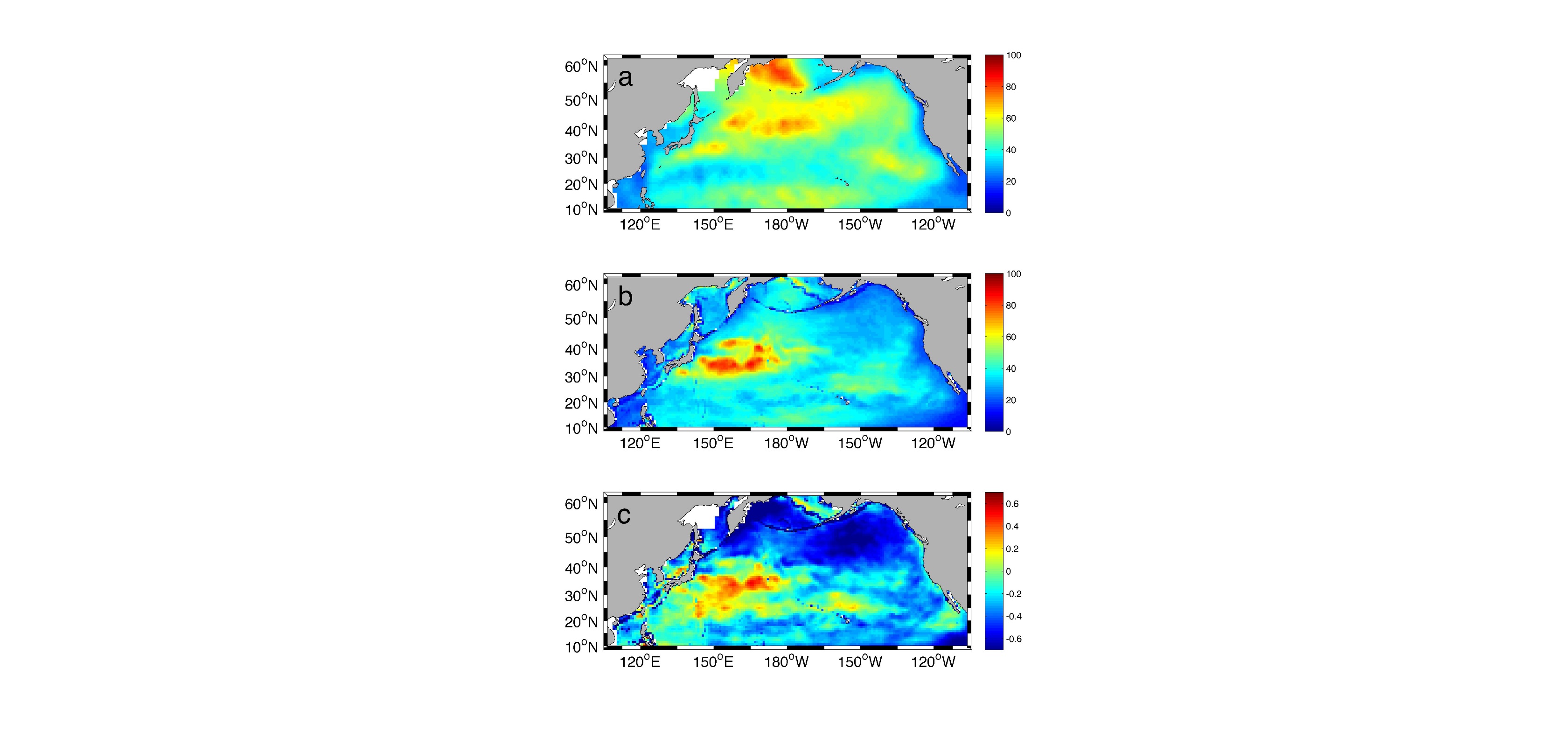


Figure S5 (a) The IFREMER/LOS Mixed Layer Depth Climatology $MLD_{obs}$, (b) the annual mean mixed layer depth $MLD_{CRCM}$ simulated in the CRCM, , and (c) their difference defined as $2(MLD_{CRCM}-MLD_{obs})/(MLD_{CRCM}+MLD_{obs})$. Here the mixed layer depth is computed as the depth at which the potential density is 0.03 kg m^-3^ denser than its value at 10 m. The maps were generated using M_Map V1.4 package for Matlab (http://www.eos.ubc.ca/~rich/map.html).


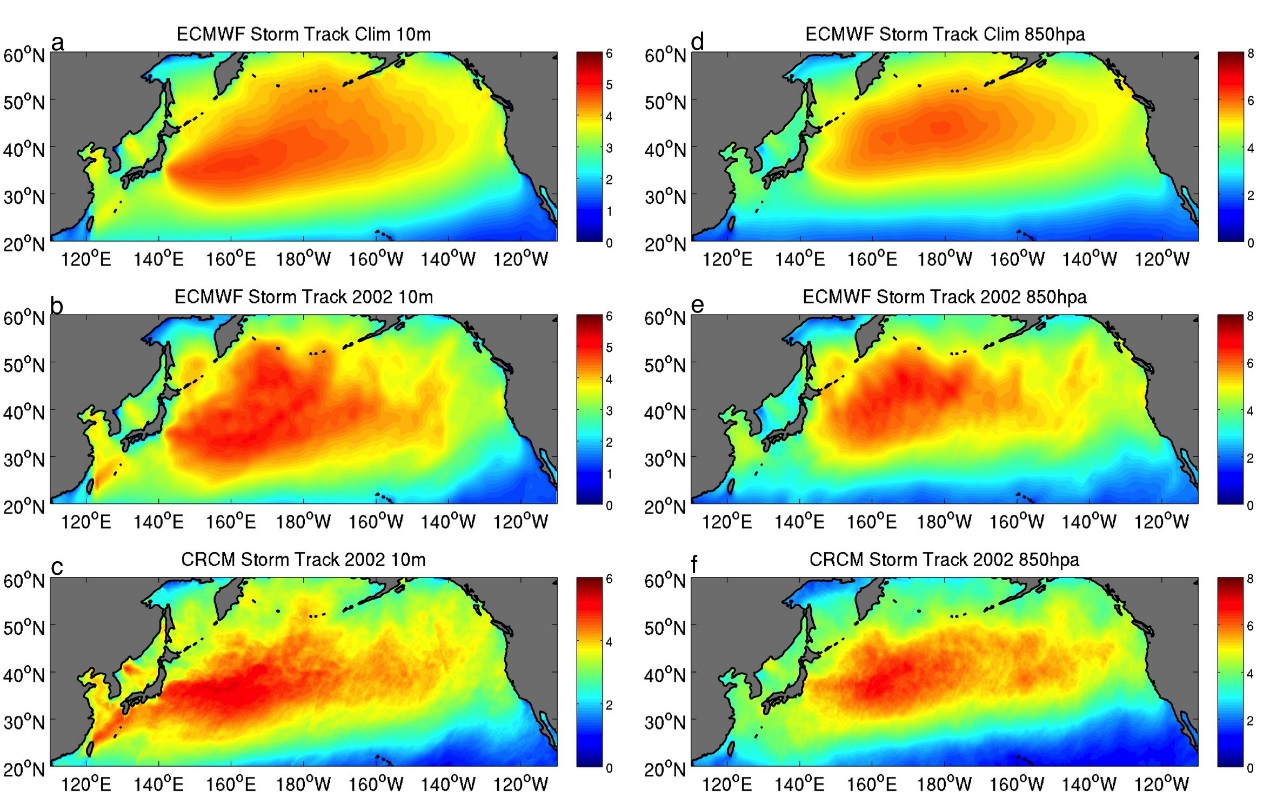


Figure S6 Winter season (December-February) sea surface (10 m height) storm track (m s^-1^) computed from the (a) ERA-Interim reanalysis in 1982-2010, (b) ERA-Interim reanalysis in 2002-2003, and (c) CRCM simulation. (d)-(f) same as (a)-(c) but for the 850-hpa storm track. Here the storm track is defined as the standard deviation of 2-8 day band-pass filtered meridional wind component. The maps were generated using M_Map V1.4 package for Matlab (http://www.eos.ubc.ca/~rich/map.html).

Figure S7 The ratio of $m_{c}$estimated using $m_{*}$=0 to those estimated using $m_{*}$=0.01 rad m^-1^ (blue) and $m_{*}$=0.02 rad m^-1^ (red), respectively.
